# Factors associated with death in newborns with gastroschisis: a retrospective cohort study from a single reference center

**DOI:** 10.1590/1806-9282.20241999

**Published:** 2025-08-08

**Authors:** Cinthia Maria Gomes da Costa Escoto Esteche, Edward Araujo, Beatriz da Costa Escoto Esteche, Larissa de Oliveira Bernardo Rodrigues, Francisco Herlânio Costa Carvalho, Lilliam Cristine Rolo

**Affiliations:** 1 Universidade Federal de São Paulo, Escola Paulista de Medicina, Department of Obstetrics – São Paulo (SP), Brazil.; 2 Centro Universitário Christus, Medical School – Fortaleza (CE), Brazil.; 3 Universidade Federal do Ceará, School of Medicine, Department of Women's, Children's and Adolescents’ Health – Fortaleza (CE), Brazil.

**Keywords:** Gastroschisis, Prenatal care, Neonatal mortality, Mechanical ventilation, Operative procedure

## Abstract

**OBJECTIVE:**

The objective of this study was to assess the risk factors for neonatal death in newborns with gastroschisis.

**METHODS:**

Hospital-based secondary analysis of a retrospective cohort study was performed in a single reference center between January 2014 and December 2023. The following variables were evaluated: maternal characteristics, obstetric aspects, habits, and labor and delivery data. Information on neonatal and hospitalization was also included.

**RESULTS:**

A total of 48 newborns with gastroschisis were delivered, with 27 (56%) being discharged from the hospital, 5 (10%) being transferred to another hospital, and 16 (33%) dying. Maternal origin was associated with neonatal death, with a 4.2 times greater chance of death if the pregnant women came from the interior. Newborns who died started prenatal care significantly earlier than the survivors. Newborns with complex gastroschisis had a 4.05 times higher risk of death, while the lack of primary closure in the first approach increased this risk by a factor of 7.0. Duration of parenteral nutrition, admission to the neonatal intensive care unit, and total hospital stay were significantly shorter in the cases of death, indicating that longer periods were associated with a 5, 6, and 6% reduction in the risk of death, respectively. On the other hand, not wearing the oxygen hood helmet increased the risk of death by 6.94 times. Newborns who spent more days on mechanical ventilation were 9% more likely to die.

**CONCLUSION:**

In conclusion, the only significant variable was the longer time on mechanical ventilation, which was associated with mortality in newborns with gastroschisis.

## INTRODUCTION

Gastroschisis is a congenital anomaly characterized by a defect in the anterior abdominal wall, usually to the right of the umbilical cord insertion. This condition results in the evisceration of the intestines and, rarely, other organs, which lie outside the abdominal cavity without an amniotic membrane covering. The pathogenesis of gastroschisis remains unclear, but it may be caused by the rupture of a physiological hernia during fetal development^1,2^.

Gastroschisis can be classified as simple, when the defect is isolated, or complex, when it presents with gastrointestinal complications or associated anomalies such as intestinal necrosis, perforation, atresia, or volvulus. Despite advances in treatment, gastroschisis is still associated with high rates of neonatal morbidity and mortality. These rates are directly related to the condition of the intestine at birth, as exposure to the toxic environment of the amniotic fluid and trauma during delivery can cause organ damage. Complications resulting from intestinal exposure include intestinal atresia, necrosis, perforation, and obstruction of the exposed intestine^1,3^.

Regarding its incidence, a study that analyzed gastroschisis data from 27 surveillance programs in 24 countries in Latin America, North America, Europe, and Iran between 1980 and 2017 found that gastroschisis occurred in 3.06 per 10,000 births, with significant variation between regions. In addition, the prevalence of gastroschisis increased in 61% of the programs included in the study during the period analyzed^4^.

Another study, using data from 74 countries, found that gastroschisis mortality is directly related to a country's level of socioeconomic development. Expanding access to prenatal ultrasound and investing in neonatal care are key to reducing this disparity, promoting prevention, and reducing mortality^5^.

To better understand the factors influencing mortality in newborns with gastroschisis, we conducted a 10-year retrospective study in a highly complex maternity hospital in the northeast of Brazil.

## METHODS

A hospital-based secondary analysis of a retrospective cohort study was performed using the Strengthening the Reporting of Observational Studies in Epidemiology (STROBE) checklist. We analyzed the medical records of all newborns with gastroschisis at the Assis Chateaubriand Maternity School, Fortaleza, Brazil, identified by ICD Q79.3 (Gastroschisis), and registered in the Live Birth Declaration in the Live Birth Information System between January 2014 and December 2023.

The study included cases of gastroschisis that were confirmed by the medical team at different stages of care, from pregnancy to the postpartum period. This approach ensured the accuracy of the diagnosis and the inclusion of well-characterized cases. However, we excluded cases involving intrauterine death, gestational age<24 weeks, and the presence of multiple major malformations to refine the study sample.

Variables predictive of outcome included maternal characteristics (age, education, and comorbidities), obstetric aspects (number of previous pregnancies, mode of delivery), habits (smoking, alcohol consumption), and labor and delivery data (birth weight, gestational age). Information on neonatal surgery (type of surgery, duration of surgery) and hospitalization (length of stay, complications) was also included.

The data was organized in tables divided into two groups: the group of newborns who died (yes) and the group of newborns who did not die (no).

The study data were collected and managed using the Research Electronic Data Capture (REDCap, Vanderbilt University, Nashville, TN, USA) electronic data collection and management tool, hosted at the Clinical Research Unit of the Federal University of Ceará (UFC). The variables were presented as mean and standard deviation, median, percentiles, minimum and maximum, frequency, and prevalence rate. Chi-square test, Wilcoxon rank sum, Fisher's exact test, and the multivariate logistic regression model were used to assess the association between variables. A significance level of 5% was used. Statistical analyses were performed using the R program. This study was approved by the Ethics Committee of the UFC (CAAE. 17811019.5.0000.5050).

## RESULTS

Between 2014 and 2023, 52 newborns with gastroschisis were delivered. After applying the inclusion and exclusion criteria, 48 newborns were selected. Of these, 27 (56%) were discharged from the hospital, 5 (10%) were transferred to another health unit, and 16 (33%) died. The maternal origin was associated with neonatal death, with a 4.2 times greater chance of death if the pregnant women came from the inland of the Ceará state. With regard to obstetric characteristics, it was observed that the newborns who died started prenatal care significantly earlier than the survivors. Apparently, late prenatal care was associated with a 21% reduction in the chance of death compared to early prenatal care. No significant association was found between maternal habits and morbidities and the death of newborns with gastroschisis. However, among the newborns who died, most mothers did not use legal or illegal drugs, and did not have comorbidities such as hypertension and gestational diabetes (Table 1).

**Table 1:** Maternal and obstetric characteristics of the studied sample.

Neonatal death				
	Yes (n=16)	No (n=32)	p-value	OR (95%CI)
Maternal age (years)	20.5±4.7^a^	21.2±4.8^a^	0.546^b^	0.97 (0.85–1.10)
Origin				
Inland	11 (69%)^a^	11 (34%)^a^	0.024^b^	4.2 (1.21–16.4)
Capital	5 (31%)	21 (66%)^a^		
Education			0.420^b^	
Elementary school	3 (19%)^a^	9 (28%)^a^		
High school	12 (75%)^a^	21 (66%)^a^		
Higher education	0 (0%)^a^	2 (6%)^a^		
Number of previous pregnancies	1.38±0.62^a^	1.72±1.25^a^	0.479^b^	0.67 (0.27–1.28)
Number of previous deliveries	0.25±0,45^a^	0.69±1.18^a^	0.156^b^	0.43 (0.12–1.09)
Number of previous abortions	0.19±0.54^a^	0.13±0.42^a^	0.741^b^	1.33 (0.31–4.97)
Gestational age at start of prenatal care (weeks)	9.3±2.8^a^	13.1±6.3^a^	0.011^b^	0.79 (0.62–0.95)
Gestational age at diagnosis of gastroschisis (weeks)	19±9^a^	22±7^a^	0.155^b^	0.95 (0.86–1.03)
Alcoholism consumption				
No	16 (100%)^a^	31 (97%)^a^	>0.999^b^	2,97 (0–NA)
Yes	0 (0%)^a^	1 (3%)^a^		
Smoking				
No	16 (100%)^a^	29 (97%)^a^	>0.999^b^	2.97 (0–NA)
Yes	0 (0%)^a^	1 (3%)^a^		
Use of illicit drugs (n=47)				
No	15 (100%)^a^	30 (94%)^a^	>0.999^b^	7.82 (0–NA)
Yes	0 (0%)^a^	2 (6%)^a^		
Hypertension				
No	16 (100%)^a^	26 (81%)^a^	0.159^b^	26.18 (0–NA)
Yes	0 (0%)^a^	6 (19%)^a^		
Gestational diabetes				
No	13 (81%)^a^	31 (97%)^a^	0.101^b^	0.14 (0.01–1.20)
Yes	3 (19%)^a^	1 (3%)^a^		

a (%); Mean±standard deviation;

b Fisher's exact test; Wilcoxon rank sum test; Chi-square test; OR: odds ratio; CI: confidence interval; NA: not available.

No significant association was found between the type of delivery and birth characteristics and death in newborns with gastroschisis. However, the predominant profile of cases that died included male newborns delivered by cesarean section without other congenital malformations, with a mean gestational age of 34.8 weeks, a birth weight of 2,181 g, and mean Apgar scores of 6.5 at the 1st and 8 at the 5th minute. It was observed that newborns with complex gastroschisis had a 4.05 times higher risk of death, while the lack of primary closure in the first approach increased this risk by a factor of 7.0. In addition, newborns who died spent significantly more days on mechanical ventilation. In contrast, the duration of parenteral nutrition, admission to the neonatal intensive care unit, and total hospital stay were significantly shorter in the cases that resulted in death, indicating that longer periods in these conditions were associated with a 5, 6, and 6% reduction in the risk of death, respectively. On the other hand, not wearing the oxygen hood helmet increased the risk of death by 6.94 times (Table 2).

**Table 2:** Delivery and postnatal characteristics of the studied sample.

Neonatal death				
	Yes (n=16)	No (n=32)	p-value	OR (95%CI)
Mode of delivery	1 (6%)^a^	1 (3%)^a^	>0.999^b^	2.07 (0.08–54.7)
No	15 (94%)^a^	31 (97%)^a^		
Yes				
Gestational age at delivery (weeks)	34.88±3.28^a^	35.81±1.57^a^	0.745^b^	0.84 (0.62–1.09)
Sex				
Female	7 (44%)^a^	16 (50%)^a^	0.683^b^	0.78 (0.23–2.60)
Male	9 (56%)^a^	16 (50%)^a^		
Birth weight (grams)	2,181±757^a^	2,381±400^a^	0.208^b^	1 (1–1)
Apgar score at the 1st minute	6.5±2.68^a^	7.31±1,96^a^	0.289^b^	0.85 (0.65–1.11)
Apgar score at the 5th minute	8.19±1.22^a^	8.69±0.59^a^	0.158^b^	0.51 (0.21–1.03)
Other congenital malformations				
No	14 (87.5%)^a^	27 (84%)^a^	>0.999^b^	1.30 (0.24–9.86)
Yes	2 (12.5%)^a^	5 (16%)^a^		
Type of gastroschisis				
Complex	6 (38%)^a^	5 (16%)^a^	0.015^b^	4.05 (0.98–17.8)
Simple	8 (50%)^a^	27 (84%)^a^		–
Primary closure in the first approach				
No	7 (44%)^a^	3 (9%)^a^	0.010^b^	7.52 (1.72–41)
Yes	9 (56%)^a^	29 (91%)^a^		
Oxygen hood				
No	9 (56%)^a^	5 (16%)^a^	0.006^b^	6.94 (1.84–29.7)
Yes	7 (44%)^a^	27 (84%)^a^		
Mechanical ventilation (days)	18±14^a^	10±9^a^	0.047^b^	1.07 (1.01–1.13)
Parenteral nutrition (days)	24±18^a^	41±29^a^	0.029^b^	0.95 (0.90–0.99)
Neonatal intensive care unit (days)	25±19^a^	48±33^a^	0.008^b^	0.94 (0.90–0.98)

a (%); Mean±standard deviation;

b Fisher's exact test; Wilcoxon rank sum test; Chi-square test; OR: odds ratio; CI: confidence interval.

Among the variables analyzed in the multivariate logistic regression, the number of days on mechanical ventilation showed a statistically significant association with neonatal death. Thus, newborns who spent more days on mechanical ventilation were 9% more likely to die (Table 3).

**Table 3:** Multivariate logistic regression of variables related to neonatal death.

Variable		OR (95%CI)	p-value*
Gestational age at delivery		1.11 (0.75–1.68)	0.613
Was primary closure performed in the first approach?			
	Yes		
	No	4.43 (0.56–48.0)	0.174
Number of approaches		0.60 (0.14–2.10)	0.448
Number of days using mechanical ventilation		1.09 (1.01–1.19)	0.028
Type of gastroschisis			
Simple			
Complex		3.32 (0.57–22.3)	0.187

OR: odds ratio; CI: confidence interval.

* binominal logistic regression.

## DISCUSSION

Early detection of gastroschisis has the potential to significantly improve prognosis, allowing referral to tertiary health services specialized in screening for related anomalies and access to multidisciplinary care. Therefore, primary care health professionals should maintain a high index of suspicion when confronted with indicators of gastroschisis during prenatal imaging examinations. In addition, it is essential that clinicians improve their ultrasound, communication, and genetic counseling skills to effectively manage these conditions^6^.

In this study, the death rate for newborns born with gastroschisis was 33%. This rate is in line with a state in a developing country like Brazil. The mortality of gastroschisis varies according to the location where the study is carried out^7^. An international multicenter study concluded that gastroschisis had a greater difference in mortality among low-income states, where a rate of 90% was observed; in middle-income states, the rate was 32%; and high-income states had a rate of 1%^5^.

This study found a 21% reduction in the risk of death from gastroschisis in newborns whose mothers started prenatal care later. This conclusion that late prenatal care is associated with lower mortality is potentially misleading, since prenatal care allows early diagnosis of gastroschisis, but it should be emphasized that diagnosis alone does not translate into neonatal survival^5^. In an Australian retrospective single-center cohort study involving 83 fetuses with gastroschisis, the mothers with this congenital anomaly were more likely living outside Sydney, had more infections in pregnancy, and were followed with a larger number of prenatal care visits, with a shorter period from the last visit to birth^8^.

A systematic review identified intra-abdominal bowel dilation, extra-abdominal bowel dilation, and polyhydramnios as prenatal ultrasound markers of complex gastroschisis. In addition, a 27% prevalence of combined intestinal complications has been identified in fetuses with complex gastroschisis, with a higher prevalence of atresia (48%), followed by necrosis (25%). In addition, a 15% prevalence of death was observed in newborns with complex gastroschisis^9^. These findings corroborate the results of this study, which showed that newborns with complex gastroschisis had a 4.05 times higher risk of death. A population-based study in the Rio Grande do Sul state, Brazil, identified maternal age <25 years as a risk factor for gastroschisis. It also found that fetuses with gastroschisis were more likely to be born weighing <2,500 g and delivered at a gestational age <37 weeks^9^. In the present study, these risk factors were not associated with an increased risk of neonatal death.

Another review of the literature on the mortality of newborns with gastroschisis between 1960 and 2020 highlighted several risk factors that have contributed to the reduction in the death rate of gastroschisis, such as the introduction of mechanical ventilation, preformed silos, parenteral nutrition, and pulmonary surfactant. This study showed that the overall risk of death from gastroschisis is less than 5%. It also suggests that determining the ideal time for delivery may be a step forward in reducing mortality. However, it highlights that survival is disproportionately lower in newborns with complex gastroschisis and in low- and middle-income countries^10^. These findings suggest that the use of parenteral nutrition, hospitalization in neonatal intensive care units, and other units reduces the likelihood of death. In this context, a retrospective study conducted from 2007 to 2017 at a tertiary referral center in Germany included 22 (66.7%) newborns with simple and 11 (33.3%) with complex gastroschisis. Primary closure of the abdominal wall defect was performed on the day of delivery in all cases^11^. These findings are consistent with the present study, which showed that failure to perform primary closure at the first attempt increased the risk of death by more than 7-fold.

A systematic review that included 3,770 newborns diagnosed with gastroschisis from 44 studies found that 1,534 of the cases were classified as simple and 288 as complex gastroschisis. The most common neonatal complications were sepsis (11.78%), necrotizing enterocolitis (2.33%), short bowel syndrome (1.37%), bowel obstruction (0.79%), and volvulus (0.23%). Immediate surgical repair was performed in 80.1% of cases, with primary closure in 69% of cases. In addition, the mean number of days of oral feeding was 33 days, while the mean number of days of hospitalization was 38 days for newborns with simple and 89 days for complex gastroschisis^12^. A retrospective observational study in Sweden with 114 pregnant women found that early gestational age at delivery, closure of abdominal wall defect in stages, intestinal atresia, and sepsis were predictors of longer hospital stay and longer duration of parenteral nutrition. Similarly, male sex was a predictor of longer hospital stay^13^. In the present study, the most common complications were sepsis and neonatal infection, although they were not associated with neonatal death.

Newborns with simple gastroschisis had a lower overall risk of infectious complications compared to newborns with complex gastroschisis. The most common complication was blood infection, which was more common in complex gastroschisis, followed by surgical site infection. Important risk factors for surgical site infection included delayed closure, silo placement, and patch closure. In addition, sutureless closure of gastroschisis had a lower risk of infectious complications compared to operative fascial closure^14^.

Early initiation of prenatal care may be associated with a higher risk of neonatal death, possibly due to selection bias: pregnant women with complications are referred earlier, but this does not guarantee the prevention of death. Further studies are needed to better understand this relationship and contribute to reducing mortality.

## CONCLUSION

In conclusion, the only significant variable was the longer time on mechanical ventilation, which was associated with mortality in newborns with gastroschisis.

## Data Availability

The datasets generated and/or analyzed during the current study are available from the corresponding author upon reasonable request.
